# Prospective study of pediatric patients presenting with idiopathic infantile nystagmus—Management and molecular diagnostics

**DOI:** 10.3389/fgene.2022.977806

**Published:** 2022-08-22

**Authors:** Nancy Aychoua, Elena Schiff, Samantha Malka, Vijay K Tailor, Hwei Wuen Chan, Ngozi Oluonye, Maria Theodorou, Mariya Moosajee

**Affiliations:** ^1^ Moorfields Eye Hospital NHS Foundation Trust, London, United Kingdom; ^2^ Institute of Ophthalmology, University College London, London, United Kingdom; ^3^ Experimental Psychology, University College London, London, United Kingdom; ^4^ Department of Ophthalmology, National University Hospital, Singapore, Singapore; ^5^ Great Ormond Street Hospital for Children NHS Foundation Trust, London, United Kingdom; ^6^ The Francis Crick Institute, London, United Kingdom

**Keywords:** FRMD7 gene, GPR143 gene, nystagmus, whole-genome sequencing, albinism

## Abstract

Idiopathic infantile nystagmus (IIN) is an inherited disorder occurring in the first 6 months of life, with no underlying retinal or neurological etiologies and is predominantly caused by mutations in the *FRMD7* gene. IIN poses a diagnostic challenge as underlying pre-symptomatic “multisystem” disorders varying from benign to life-threatening should first be ruled out before nystagmus can be labeled as idiopathic. A multidisciplinary approach including multimodal ocular investigations and next-generation sequencing with whole-genome sequencing (WGS) or targeted gene panel testing is required to delineate the exact etiology. We report the clinical and genetic outcomes of 22 patients, from 22 unrelated families of diverse ethnicities, with IIN seen in the ocular genetics service at Moorfields Eye Hospital NHS Foundation Trust between 2016 and 2022. Thirty-six percent (8/22) received a confirmed molecular diagnosis with eight mutations identified in two genes (seven in *FRMD7* including one novel variant c.706_707del; p. [Lys236Alafs*66], and one in *GPR143*). This study expands the mutational spectrum of IIN and highlights the significant role of an integrated care pathway and broader panel testing in excluding underlying pathologies.

## Introduction

Anomalous rhythmic involuntary oscillation of the eyes occurring in the first 6 months of life is a characteristic trait of infantile nystagmus (IN; MIM# 301700) (Casteels et al., 1992). This can be idiopathic or associated with a variety of ocular disorders, such as albinism, retinal diseases, and optic nerve hypoplasia, or it may be a feature of neurological diseases, for example, spinocerebellar ataxia, glioma, and structural brain malformations (Thomas et al., 2017). It is important to differentiate between idiopathic and disease-associated nystagmus through the use of appropriate investigations as the outcome affects treatment modalities and visual prognosis.

Idiopathic infantile nystagmus (IIN) has no other underlying ocular or neurological features. Strabismus is not common, and good stereopsis is often present, with visual acuities of 0.3 LogMAR or better (Sarvananthan et al., 2009; Papageorgiou et al., 2014). It is usually bilateral, conjugate, occurring in a horizontal plane, and is of either a pendular or jerk waveform with an accelerating slow phase, but it may also rarely appear as primarily vertical or even torsional nystagmus (Papageorgiou et al., 2014). IIN has an incidence in the UK of 1.9 per 10,000, accounting for approximately 8% of all nystagmus cases which are estimated to be 24 per 10,000 of the population (Sarvananthan et al., 2009).

The clinical diagnostic pathway of patients with infantile nystagmus visiting the ocular genetic service at Moorfields Eye Hospital NHS Foundation Trust, which oversees the care of the largest number of genetic eye disease patients of any one site in the United Kingdom, involves multidisciplinary input and extensive phenotyping. All patients undergo detailed ophthalmic, systemic, and neurological examinations, and, where possible, spectral-domain optical coherence tomography (SD-OCT), electroretinograms (ERGs), visual-evoked potentials (VEPs), eye movement recordings (EMRs), and, in some instances, magnetic resonance imaging (MRI) of the brain, if indicated.

When seeking a diagnosis, the exclusion of other ocular and neurological diseases is a key element for establishing the diagnosis of idiopathic infantile nystagmus. A common presenting sign of early-onset retinal dystrophies is nystagmus, and in many cases, a glance at the fundus can appear normal. SD-OCT of the macula is helpful in identifying foveal hypoplasia and other retinal abnormalities (Self et al., 2020). Electrodiagnostic tests including ERG and VEP are extremely useful, especially in young children, in assessing the integrity of the retina and visual pathway as they aid in identification of post-retinal pathology such as intracranial chiasmal misrouting seen in albinism (Self et al., 2020). Oscillatory eye movements that are not visible to the naked eye or occur transiently can be objectively visualized through eye movement recordings (EMRs). They can reveal the underlying nystagmus waveform that distinguishes various types of nystagmus and saccadic oscillations (Self et al., 2020).

After extensive and thorough ocular phenotyping, which focuses on ruling out alternative diagnoses, genetic testing plays a significant role in the diagnostic trajectory of patients presenting with nystagmus. Targeted gene panels exist for nystagmus, albinism, complex strabismus, and retinal dystrophies, and in some patients, for example, younger children where other investigations such as electrodiagnostics have been inconclusive due to compliance, combinations of these may be required in order to not overlook a molecular cause (O’Gorman et al., 2019). To date, only *FRMD7* (MIM# 300628) and *GPR143* (MIM# 300808) have been reported to be responsible for causing IIN. *GPR143* is predominantly associated with ocular albinism, type I (MIM# 300500) (Almoallem et al., 2015) (Tarpey et al., 2006).

The FERM domain-containing 7 (*FRMD7*) gene is located on chromosome Xq26.2 and comprises 12 exons, encoding a 714-amino acid protein which is a member of the plasma membrane cytoskeleton coupling proteins. Variants in *FRMD7* account for approximately 70% of known IIN cases (Tarpey et al., 2006) (Salman et al., 2022). Though the function of *FRMD7* is not yet fully understood, it is expressed in both the developing neural retina and ocular motor structures such as the cerebellum and vestibular-optokinetic system, playing a role in the control of eye movement and gaze stability, as was recently confirmed by Salman *et al* in the starburst amacrine cells of mutant Frmd7 ^tm1a^ and Frmd7 ^tm1b^ mouse models (Almoallem et al., 2015) (Tarpey et al., 2006) (Choi et al., 2018) (Li et al., 2014) (Pu et al., 2019).

The G protein-coupled receptor 143 (*GPR143*) gene which is also located on chromosome Xp22.2 comprises nine exons and encodes a protein of 404 amino acids. It is expressed at high levels in the retina, including the retinal pigment epithelium (RPE) and melanocytes, with weaker expression in the brain and adrenal glands. Mutations involving this gene are most commonly known to cause ocular albinism (OA; MIM# 300500), invariably characterized by infantile nystagmus (Bassi et al., 1995). However, variants in *GPR143* have also been found to cause IIN without the classical manifestations of OA, which include chiasmal misrouting, fundus hypopigmentation, iris transillumination, and gray optic nerves with or without optic nerve hypoplasia (Liu et al., 2007); (Peng et al., 2009).

The aim of this study is to expand the molecular and clinical spectrum of idiopathic infantile nystagmus in a prospective cohort of patients presenting with infantile nystagmus. This study highlights the importance of accurate phenotyping and genotyping to ensure the correct diagnosis is made with informed genetic counselling, family planning, and access to suitable treatments and trials for patients and their families.

## Methods

### Editorial policies and ethical considerations

The study had relevant local and national research ethics committee approvals (MEH and Northwest London Research Ethics Committee) and conformed to the tenets of the Declaration of Helsinki. Patients and relatives gave written informed consent for genetic testing, through either the Genetic Study of Inherited Eye Disease (REC reference 12/LO/0141) or the Genomics England 100,000 Genomes project (REC reference 14/EE/1112).

This was a prospective study of patients presenting with infantile nystagmus to the ocular genetics service at Moorfields Eye Hospital NHS Foundation Trust (MEH), London, United Kingdom, between the 1st of January 2016 and the 21st of June 2022. Patients who had clinical features of idiopathic infantile nystagmus, without any syndromic or systemic manifestations, were recruited into this study.

### Clinical assessment

Data collected included full medical history, family history, developmental pediatric assessment, best corrected visual acuity (BCVA), eye movement recordings (EMR) of nystagmus, slit lamp biomicroscopy of the anterior segment, and fundus examination. Best corrected visual acuity (BCVA) was measured using LogMAR or Cardiff cards for preverbal children up to 36 months of age. Visual acuities were translated into LogMAR acuities using a LogMAR conversion table (Hargadon et al., 2010). Where the child was unable to perform an accurate acuity test, descriptive visual behavior was documented. Spectral-domain optical coherence tomography (SD-OCT) was performed using the OCT SPECTRALIS® (Heidelberg Engineering GmbH, Heidelberg, Germany). When clinically relevant and where possible, electroretinograms and multichannel flash VEP were performed according to the international Society for Clinical Electrophysiology of Vision (ISCEV) standards at the Department of Electrophysiology, MEH (Odom et al., 2016). When indicated, an MRI brain was also undertaken. The MRI sequences obtained varied depending on symptomatology and clinical questions at the time of the initial presentation of patients, and therefore, a range of protocols were applied; however, all had sagittal and axial T1-weighted, axial T2-weighted, and coronal STIR imaging, allowing assessment of the orbits, the visual pathway, the hypothalamic–pituitary axis, the cerebral midline, and the cortex.

### Genetic testing

Molecular analysis was performed using either targeted gene panel testing or whole-genome sequencing (WGS). Clinical exome panel testing was performed through the Rare & Inherited Disease Genomic Laboratory at Great Ormond Street Hospital (London, UK) with a virtual nystagmus and albinism gene panel applied (http://www.labs.gosh.nhs.uk/media/764794/oculome_v8.pdf). WGS was performed as part of the UK Genomics England 100,000 Genomes Project, for which results were reviewed by multidisciplinary teams (including molecular biologists and clinical geneticists, as well as the ophthalmology specialist managing the family), to confirm the variant pathogenicity prevalence in the publicly available genome databases, the clinical phenotype, and the mode of inheritance before the molecular diagnosis was established. The datasets (variants) generated for this study were submitted to ClinVar (https://www.ncbi.nlm.nih.gov/clinvar/). Genetic samples of parents and/or additional family members were obtained where available for co-segregation and to assist with assessment of variant pathogenicity.

## Results

### Clinical findings

**FIGURE 1:**
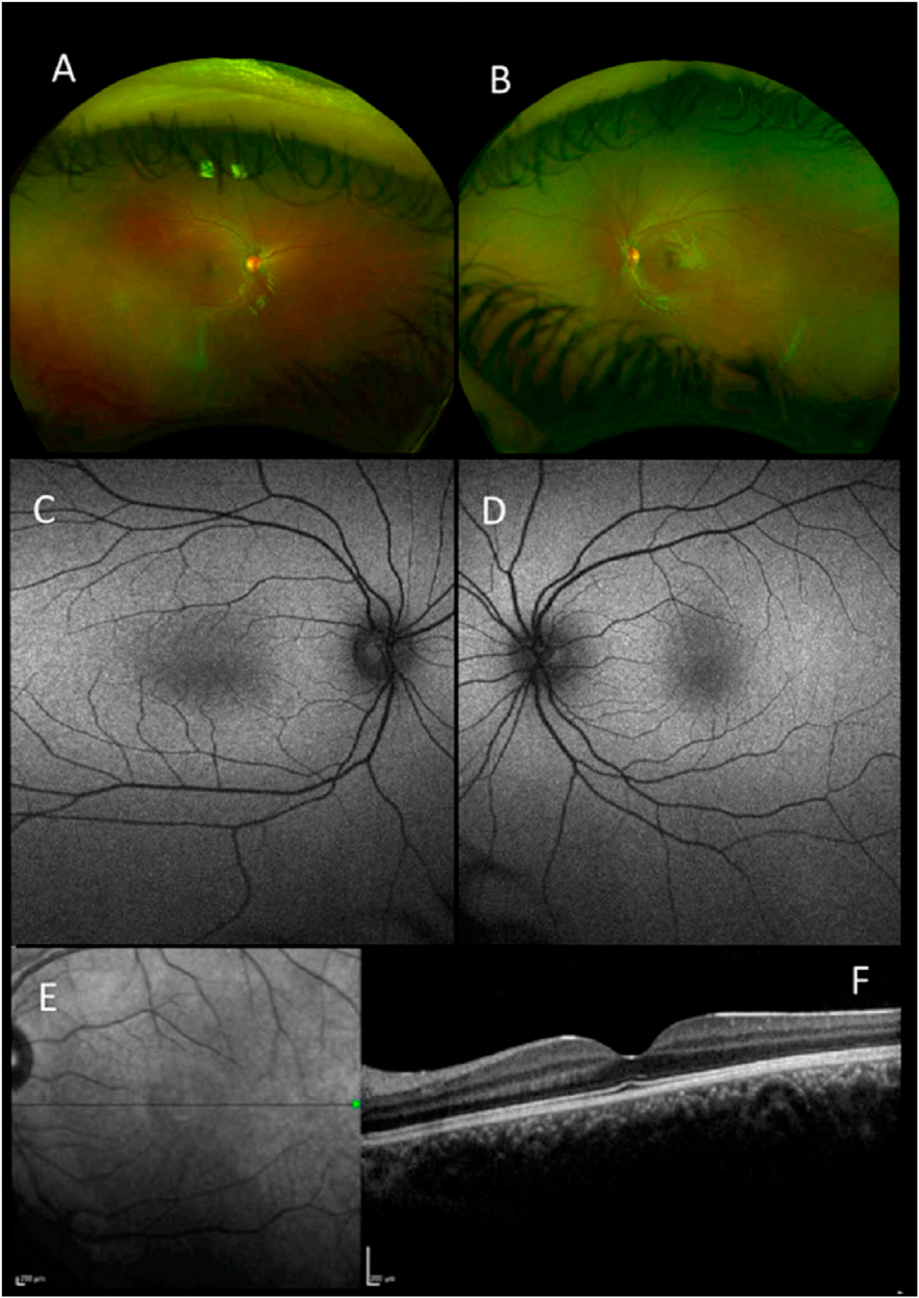
Retinal imaging-pseudocolor **(A,B)**, autofluorescence (Optos) **(C,D)**, near-infrared scanning laser ophthalmoscope (SLO) fundus image **(E)** and foveal optical coherence tomography (OCT) (Heidelberg Spectralis) **(F)** all for patient 1 with a mutation in FRMD7.

The clinical findings of our cohort of 22 patients are summarized in Table 1. The study included 13 male (59%) and nine female (41%) unrelated probands. In six patients, additional family members with comparable complaints of nystagmus were reported. There was no report of consanguinity in any families. The ethnicity of patients was divided into six White British (27.3%), one White other (4.5%), four other ethnicities (18.2%), one Black other (4.5%), and 10 were not stated (45.5%). The mean age of onset of nystagmus was 2.7 months. Mean BCVA was 0.4 [± 0.18] LogMAR (ranging from 0.1 to 0.7 LogMAR). SD-OCT images were obtained in 19 patients. SD-OCT images were not possible in three patients due to poor cooperation. There were five patients (26%) with foveal hypoplasia, 11 (58%) with normal foveal architecture, and three (16%) with poor quality images (Figure 1). Nineteen patients (86%) underwent electrodiagnostic testing (both ERG and VEP). None had intracranial chiasmal misrouting or showed any signs of retinal pathology.

**TABLE 1:** Summary of demographics and clinical features of all idiopathic infantile nystagmus (IIN) patients presenting to Moorfields Eye Hospital NHS Foundation Trust (MEH) Genetics Service 2016–2022.

ID	GC num	Ethnicity	Gender	Age (y)	BCVA OD	BCVA OS	Nystagmus-waveform	OCT	Age of onset	Family history?	Genetic testing performed	Molecular diagnosis	Gene
1	26264	Other: Other	Male	14	0.5	0.5	Pendular	Normal	Birth	Y	WGS	Solved	*FRMD7*
2	26339	White: British	Male	4	0.6	0.7	Waveform not described	-	Birth	Y	WGS	NPF	-
3	26362	Not Stated	Male	7	0.2	0.2	Horizontal	Normal	Birth	N	Oculome	NPF	-
4	26675	White: Other	Male	6	0.3	0.5	Pendular	Foveal hypoplasia	Birth	N	Oculome	NPF	-
5	27015	Other: Other	Female	3	0.5	0.6	Pendular	Normal	Birth	N	Oculome	NPF	-
6	27071	Not Stated	Female	3	0.5	0.5	Pendular	Normal	Birth	N	Oculome	NPF	-
7	27269	Not Stated	Male	3	0.4	0.4	Pendular	poor quality	Birth	N	Oculome	Solved	*FRMD7*
8	27300	Not Stated	Male	4	0.3	0.3	Pendular	poor quality	Birth	N	Oculome	Solved	*FRMD7*
9	27324	Other: Other	Male	3	0.1	0.2	Horizontal	poor quality	Birth	N	Oculome	NPF	-
10	27923	Not Stated	Male	10	0.4	0.6	Horizontal	Normal	Birth	Y	Oculome	NPF	-
11	28353	Not Stated	Female	2	0.5	0.5	Pendular	-	Birth	N	Oculome	NPF	-
12	10282	White: British	Female	48	0.2	0.2	Waveform not described	-	Birth	Y	WGS	Solved	*FRMD7*
13	26131	Mixed: Other	Female	42	0.1	0.1	Waveform not described	Normal	Birth	Y	WGS	Solved	*FRMD7*
14	18284	White: British	Male	5	0.2	0.2	Periodically alternating	Normal	Birth	Y	WGS	Solved	*FRMD7*
15	7449	White: British	Male	25	0.5	0.5	Waveform not described	Normal	Birth	N	Oculome	NPF	-
16	41673	Other: Other	Female	40	0.3	0.3	Waveform not described	Foveal hypoplasia	Birth	N	Oculome	NPF	-
17	41308	Not Stated	Female	52	0.5	0.2	Pendular	Foveal hypoplasia	3	N	Oculome	NPF	-
18	42563	Not Stated	Female	19	0.6	0.5	Pendular	Normal	2	N	Oculome	NPF	-
19	27482	White: British	Female	24	0.2	0.2	Horizontal	Normal	Birth	N	WGS	NPF	-
20	43652	White: British	Male	23	0.7	0.7	Horizontal	Foveal hypoplasia	Birth	N	Oculome	NPF	-
21	28575	Not Stated	Male	1	-	-	Waveform not described	Normal	Birth	N	Blueprint	Solved	*GPR143*
22	28978	Not Stated	Male	6	0.3	0.4	Horizonal	Foveal hypoplasia	Birth	N	Oculome	Solved	*FRMD7*

### Molecular findings

**FIGURE 2:**
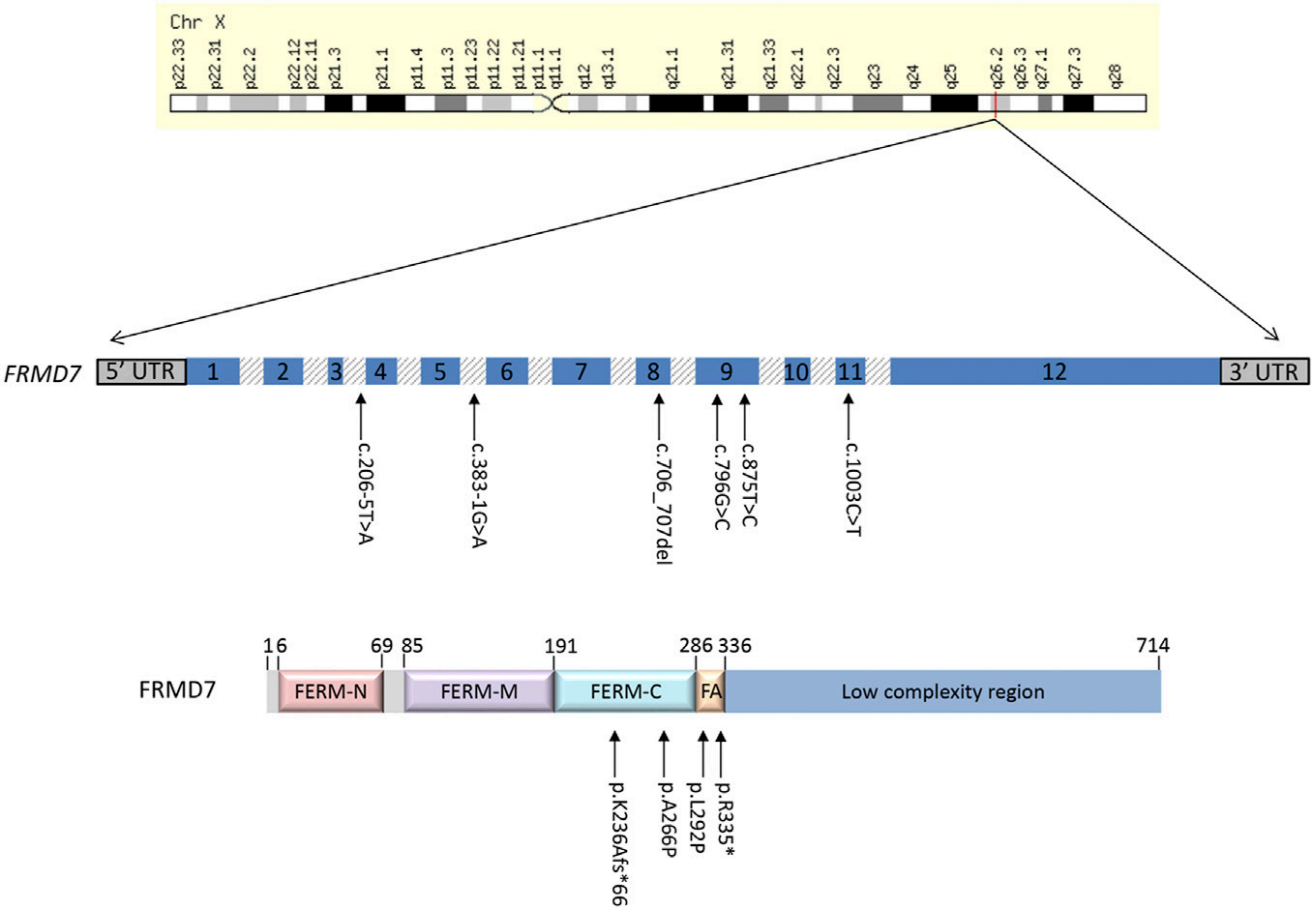
Schematic representation of the *FRMD7* gene and protein highlighting mutations (arrows) in patients with idiopathic infantile nystagmus (IIN). Exons are indicated with numbered boxes, and introns, which are not in proportion, appear shaded in gray.

**FIGURE 3:**
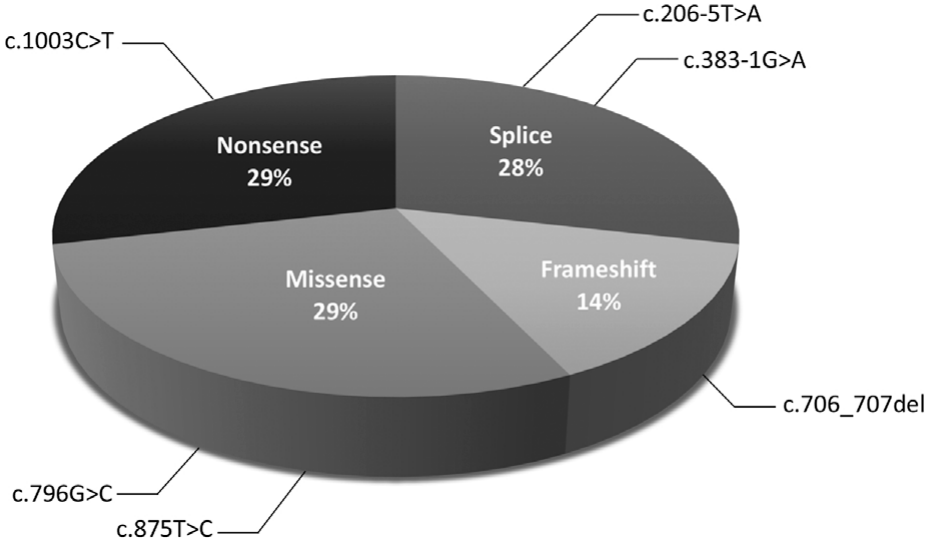
Distribution of mutations in *FRMD7* in idiopathic infantile nystagmus (IIN).

The molecular findings are summarized in Table 2. Pathogenic variants in the X-linked genes *FRMD7* (Figure 2) and *GPR143* were detected in eight out of 22 (36%) unrelated patients who satisfied the inclusion criteria of IIN. Segregation of the variants was performed in all parents except in patient 13. All *FRMD7* variants detected were classified as either likely pathogenic or pathogenic by the American College of Medical Genetics and Genomics (ACMG) guidelines (Richards et al., 2015). One variant was novel, a frameshift c.706_707del p. (Lys236Alafs*66) in exon 7. This frameshift mutation is predicted to result in a truncated protein and is located in the conserved N terminal F3-FERM domain of the protein, which is one of the two FRMD7 domains with a cluster of variants (Watkins et al., 2012). The additional four likely pathogenic *FRMD7* variants have all been previously reported to cause IIN; a likely pathogenic splice variant c.383-1G>A (Thomas et al., 2017; Jackson et al., 2020), two likely pathogenic missense variants c.796G>C p. (Ala266Pro) (Sarvananthan et al., 2009; Papageorgiou et al., 2014), and c.875T>C p. (Leu292Pro) (Thomas et al., 2017; Self et al., 2020) and a nonsense variant c.1003C>T p. (Arg335*) (Tarpey et al., 2006; Zhang et al., 2007; Watkins et al., 2012) (Figure 3).

**TABLE 2:** List of variants in molecularly confirmed idiopathic infantile nystagmus (IIN).

Family ID	Gene	Transcript	Inheritance	Nucleotide change	Protein change	Mutation type	Variant classification	Polyphen-2	CADD v.1.6	gnomAD	References
21	*GPR143*	NM_000273.3	XLR	c.733C>T	p. (Arg245*)	Nonsense	Pathogenic	NA	36	Absent	Schnur et al., 1998 PMID: 9529334; Khan et al., 2016 PMID: 26785811
12	*FRMD7*	NM_194277.3	XLR	c.206–5T>A	-	Splice	Likely Pathogenic	NA	23.6		Jackson et al., 2020 PMID: 32830442
1	*FRMD7*	NM_194277.3	XLR	c.383-1G>A	-	Splice	Pathogenic	NA	33	Absent	Jackson et al., 2020 PMID: 32830442
8	*FRMD7*	NM_194277.3	XLR	c.706_707del	p. (Lys236Alafs*66)	Frameshift	Pathogenic	NA	36	Absent	Novel
7	*FRMD7*	NM_194277.3	XLR	c.796G>C	p. (Ala266Pro)	Missense	Likely Pathogenic	probably damaging	24.6	Absent	Tarpey et al., 2006 PMID: 17013395
13	*FRMD7*	NM_194277.3	XLR	c.875T>C	p. (Leu292Pro)	Missense	Likely Pathogenic	probably damaging	26.6	0.002246 in S Asians only	Jackson et al., 2020 PMID: 32830442
14	*FRMD7*	NM_194277.3	XLR	c.1003C>T	p. (Arg335*)	Nonsense	Pathogenic	NA	35	Absent	Tarpey et al., 2006 PMID: 17013395; Zhang et al., 2007 PMID: 17893669; Jackson et al., 2020 PMID: 32830442
22	*FRMD7*	NM_194277.3	XLR	c.1003C>T	p. (Arg335*)	Nonsense	Pathogenic	NA	35	0.0000122 in Europeans only	Tarpey et al., 2006 PMID: 17013395; Thomas et al., 2011 PMID: 21303855; Guo et al., 2014 PMID: 24513357

Two additional patients had likely pathogenic splice variants in *FRMD7*, c.383-1G>A and c.206–5T>A. The latter variant has previously been predicted to result in obliteration of the splice acceptor site in intron 3 (Thomas et al., 2014). It segregated with the affected father of proband ID#12 GC10282-2.

A pathogenic nonsense mutation c.733C>T p. (Arg245*) was identified in *GPR143* in proband ID# 21. This variant has been previously reported in two brothers with features of ocular albinism, including ocular hypopigmentation and developmental delay and facial dysmorphia (Schnur et al., 1998) (Khan et al., 2016). Two Korean brothers in a second reported family only show features of congenital nystagmus (Kim et al., 2016).

## Discussion

In this study, 22 subjects were clinically diagnosed with idiopathic infantile nystagmus, of which 36% received a genetic diagnosis. The most prevalent causative gene was *FRMD7*, although a variant in *GPR143* was also identified. The percentage of solved cases in our cohort is in line with that of previous studies. AlMoallem *et al* found that 22.4% of probands harbored variants in *FRMD7* but none involving *GPR143* (Almoallem et al., 2015). Choi et al. reported a 35% molecular diagnostic rate of *FRMD7*-associated infantile nystagmus syndrome in Korean probands (Choi et al., 2018).

*GPR143* mutations have been previously identified in patients with X-linked IIN without any classical phenotype of OA (Zhou et al., 2008) (Peng et al., 2009). Electrophysiology in Proband ID#21, with c.733C>T p. (Arg245*), concluded that flash VEPs showed no evidence of crossed asymmetry and the findings were not consistent with albinism. The flash ERGs did not reveal any evidence of generalized rod or cone system dysfunction. Interestingly, Zou *et al* describe a similar case with c.793C>T, p. (R265X) variant in a Chinese family with ocular albinism and normal VEPs but suggest that the chiasmal misrouting was possibly so mild that it was likely missed by electrophysiology (Zou et al., 2017). In addition, Kim *et al.* describe the same variant in two Korean brothers with congenital nystagmus, but no electrophysiology was reported (Kim et al., 2016).

It is important to underline that young children with inherited retinal diseases presenting with nystagmus often have normal retinal findings on fundoscopy and may be misdiagnosed with IIN. In addition, those with albinism may not show fulminant features, and hence electrophysiology is a key investigation that can help detect early clinical signs of disease (Simon et al., 1984; Neveu et al., 2022). The importance of genetic testing to determine the molecular diagnosis cannot be emphasized enough, especially, in young children with poor cooperation as it helps exclude progressive inherited retinal disorders or albinism. A molecular diagnosis provides insights into the potential co-morbidities and signposts the most appropriate multidisciplinary team that is required to optimize the patient care pathway. In general, OCT imaging can be difficult to perform in very young children due to compliance, and despite the non-invasive nature of pediatric ERGs/VEPs (which do not require any anesthesia, sedation, or mydriasis and are relatively quick) (Self et al., 2020), often outcomes are unreliable and inconclusive in children (Thomas et al., 2017; O’Gorman et al., 2019). From our cohort, three patients had an inconclusive ERG, but genetic testing yielded a positive result in one of them. Electrophysiology results, particularly the pattern ERG, in idiopathic nystagmus can be inconclusive due to eye movement artifacts on the recording, while VEP from hemisphere asymmetries that are not consistent with albinism or hemisphere asymmetries that are inconsistent between eyes, often due to strabismus. Within this cohort, only 12 patients (55%) had their ethnicity recorded, out of which only four patients received a molecular diagnosis. This small sample size is unable to provide any meaningful conclusions regarding the diagnostic yield in patients with different ethnic backgrounds.

Of the fourteen patients in whom a molecular diagnosis was not confirmed, 12 had the oculome with a targeted albinism and nystagmus gene panel and two had WGS. It is possible that these probands may have deep intronic variants, missed by the filtering methods used or potential variants in, as yet, unidentified genes. Further interrogation of the WGS data and further research may lead to identification of these hitherto evasive variants**.**

## Conclusion

In summary, we have demonstrated that IIN has a strong genetic basis with a 36% molecular diagnostic rate. Variants involving *FRMD7* are the most common cause of IIN. We report a novel *FRMD7* variant herein, expanding the already known gene mutation spectrum of idiopathic infantile nystagmus. Only when neurological and retinal diseases are promptly excluded, infantile nystagmus can be safely diagnosed as idiopathic. To safeguard efficiency, accuracy, and counteract any diagnostic delay, pediatric and ophthalmic clinical assessments, supported by ocular imaging, electrophysiology, eye movement recordings, and genetic testing, are indispensable in ensuring a complete and succinct clinical diagnostic pathway. Implementation of a broader gene panel including nystagmus, albinism, and retinal genes may be more appropriate for correctly diagnosing idiopathic infantile nystagmus or related masqueraders, which may have serious life-threatening implications if overlooked.

## Data Availability

The original contributions presented in the study are included in the article/Supplementary Material; further inquiries can be directed to the corresponding author.

## References

[B1] Almoallem B. Bauwens M. Walraedt S. Delbeke P. De Zaeytijd J. Kestelyn P. (2015). Novel FRMD7 mutations and genomic rearrangement expand the molecular pathogenesis of X-linked idiopathic infantile nystagmus. Invest. Ophthalmol. Vis. Sci. 56 (3), 1701–1710. 10.1167/iovs.14-15938 25678693

[B2] Bassi M. T. Schiaffino M. V. Renieri A. De Nigris F. Galli L. Bruttini M. (1995). Cloning of the gene for ocular albinism type 1 from the distal short arm of the X chromosome. Nat. Genet. 10 (1), 13–19. 10.1038/ng0595-13 7647783

[B3] Casteels I. Harris C. M. Shawkat F. Taylor D. (1992). Nystagmus in infancy. Br. J. Ophthalmol. 76 (7), 434–437. 10.1136/bjo.76.7.434 1627515 PMC504306

[B4] Choi J. H. Jung J. H. Oh E. H. Shin J. H. Kim H. S. Seo J. H. (2018). Genotype and phenotype spectrum of FRMD7-associated infantile nystagmus syndrome. Invest. Ophthalmol. Vis. Sci. 59 (7), 3181–3188. 10.1167/iovs.18-24207 30025138

[B5] Hargadon D. D. Wood J. Twelker J. D. Harvey E. M. Dobson V. (2010). Recognition acuity, grating acuity, contrast sensitivity, and visual fields in 6-year-old children. Arch. Ophthalmol. 128 (1), 70–74. 10.1001/archophthalmol.2009.343 20065220

[B6] Jackson D. Malka S. Harding P. Palma J. Dunbar H. Moosajee M. (2020). Molecular diagnostic challenges for non‐retinal developmental eye disorders in the United Kingdom. Am. J. Med. Genet. C Semin. Med. Genet. 184 (3), 578–589. 10.1002/ajmg.c.31837 32830442 PMC8432170

[B7] Khan A. O. Tamimi M. Lenzner S. Bolz H. J. (2016). Hermansky-Pudlak syndrome genes are frequently mutated in patients with albinism from the Arabian Peninsula. Clin. Genet. 90 (1), 96–98. 10.1111/cge.12715 26785811

[B8] Kim U. S. Cho E. Kim H. J. (2016). A novel nonsense mutation of GPR143 gene in a Korean kindred with X-linked congenital nystagmus. Int. J. Ophthalmol. 9 (9), 1367–1370. 10.18240/ijo.2016.09.25 27672609 PMC5028679

[B9] Li Y. Pu J. Zhang B. (2014). Expression of a novel splice variant of FRMD7 in developing human fetal brains that is upregulated upon the differentiation of NT2 cells. Exp. Ther. Med. 8 (4), 1131–1136. 10.3892/etm.2014.1916 25187810 PMC4151643

[B10] Liu J. Y. Ren X. Yang X. Guo T. Yao Q. Li L. (2007). Identification of a novel GPR143 mutation in a large Chinese family with congenital nystagmus as the most prominent and consistent manifestation. J. Hum. Genet. 52 (6), 565–570. 10.1007/s10038-007-0152-3 17516023

[B11] Neveu M. M. Padhy S. K. Ramamurthy S. Takkar B. Jalali S. Cp D. (2022). Ophthalmological manifestations of oculocutaneous and ocular albinism: Current perspectives. Clin. Ophthalmol. 16, 1569–1587. 10.2147/OPTH.S329282 35637898 PMC9148211

[B12] Odom J. V. Bach M. Brigell M. Holder G. E. McCulloch D. L. Mizota A. (2016). ISCEV standard for clinical visual evoked potentials: (2016 update). Doc. Ophthalmol. 133 (1), 1–9. 10.1007/s10633-016-9553-y 27443562

[B13] O’Gorman L. Norman C. S. Michaels L. Newall T. Crosby A. H. Mattocks C. (2019). A small gene sequencing panel realises a high diagnostic rate in patients with congenital nystagmus following basic phenotyping. Sci. Rep. 9 (1), 13229. 10.1038/s41598-019-49368-7 31519934 PMC6744446

[B14] Papageorgiou E. McLean R. J. Gottlob I. (2014). Nystagmus in childhood. Pediatr. Neonatol. 55 (5), 341–351. 10.1016/j.pedneo.2014.02.007 25086850

[B15] Peng Y. Meng Y. Wang Z. Qin M. Li X. Dian Y. (2009). A novel GPR143 duplication mutation in a Chinese family with X-linked congenital nystagmus. Mol. Vis. 15, 810–814. 19390656 PMC2671585

[B16] Pu J. Dai S. Gao T. Hu J. Fang Y. Zheng R. (2019). Nystagmus-related FRMD7 gene influences the maturation and complexities of neuronal processes in human neurons. Brain Behav. 9 (12), e01473. 10.1002/brb3.1473 31743612 PMC6908866

[B17] Richards S. Aziz N. Bale S. Bick D. Das S. Gastier-Foster J. (2015). Standards and guidelines for the interpretation of sequence variants: A joint consensus recommendation of the American College of medical genetics and Genomics and the association for molecular pathology. Genet. Med. 17 (5), 405–424. 10.1038/gim.2015.30 25741868 PMC4544753

[B18] Salman A. Hutton S. B. Newall T. Scott J. A. Griffiths H. L. Lee H. (2022). Characterization of the FRMD7 knock-out mice generated by the EUCOMM/COMP repository as a model for idiopathic infantile nystagmus (IIN). Genes (Basel) 11 (10), E1157. 10.3390/genes11101157 PMC7601595 33007925

[B19] Sarvananthan N. Surendran M. Roberts E. O. Jain S. Thomas S. Shah N. (2009). The prevalence of nystagmus: The leicestershire nystagmus survey. Invest. Ophthalmol. Vis. Sci. 50 (11), 5201–5206. 10.1167/iovs.09-3486 19458336

[B20] Schnur R. E. Gao M. Wick P. A. Keller M. Benke P. J. Edwards M. J. (1998). OA1 mutations and deletions in X-linked ocular albinism. Am. J. Hum. Genet. 62 (4), 800–809. 10.1086/301776 9529334 PMC1377018

[B21] Self J. E. Dunn M. K. Erichsen J. T. Gottlob I. Griffiths H. J. Harris C. (2020). Management of nystagmus in children: A review of the literature and current practice in UK specialist services. Eye (Basingstoke) (9), 1515–1534. 10.1038/s41433-019-0741-3 PMC7608566 31919431

[B22] Simon J. W. Kandel G. L. Krohel G. B. Nelsen P. T. (1984). Albinotic characteristics in congenital nystagmus. Am. J. Ophthalmol. 97 (3), 320–327. 10.1016/0002-9394(84)90630-5 6702969

[B23] Tarpey P. Thomas S. Sarvananthan N. Mallya U. Lisgo S. Talbot C. J. (2006). Mutations in FRMD7, a newly identified member of the FERM family, cause X-linked idiopathic congenital nystagmus. Nat. Genet. 38 (11), 1242–1244. 10.1038/ng1893 17013395 PMC2592600

[B24] Thomas M. G. Crosier M. Lindsay S. Kumar A. Araki M. Leroy B. P. (2014). Abnormal retinal development associated with FRMD7 mutations. Hum. Mol. Genet. 23 (15), 4086–4093. 10.1093/hmg/ddu122 24688117 PMC4082370

[B25] Thomas M. G. Maconachie G. D. E. Sheth V. McLean R. J. Gottlob I. (2017). Development and clinical utility of a novel diagnostic nystagmus gene panel using targeted next-generation sequencing. Eur. J. Hum. Genet. 25 (6), 725–734. 10.1038/ejhg.2017.44 28378818 PMC5477371

[B26] Watkins R. J. Thomas M. G. Talbot C. J. Gottlob I. Shackleton S. (2012). The role of FRMD7 in idiopathic infantile nystagmus. J. Ophthalmol. 2012, 460956. 10.1155/2012/460956 21904664 PMC3163398

[B27] Zhang B. Liu Z. Zhao G. Xie X. Yin X. Hu Z. (2007). Novel mutations of the FRMD7 gene in X-linked congenital motor nystagmus. Mol. Vis. 13, 1674–1679. 17893669

[B28] Zhou P. Wang Z. Zhang J. Hu L. Kong X. (2008). Identification of a novel GPR143 deletion in a Chinese family with X-linked congenital nystagmus. Mol. Vis. 14, 1015–1019. 18523664 PMC2408774

[B29] Zou X. Li H. Yang L. Sun Z. Yuan Z. Li H. (2017). Molecular genetic and clinical evaluation of three Chinese families with X-linked ocular albinism. Sci. Rep. 7, 33713. 10.1038/srep33713 28211458 PMC5314354

